# Supplementary material for: Profiling the most elderly parkinson’s disease patients: Does age or disease duration matter?
Source: PLoS One. 2021 Dec 22;16(12):e0261302. doi: 10.1371/journal.pone.0261302 (PMC8694485; doi:10.1371/journal.pone.0261302)
Supplement: S1 Table — TD, tremor-dominant; PIGD, postural instability/gait difficulty; UPDRS, Unified Parkinson’s Disease Rating Scale; H&Y-S, Hoehn & Yahr staging; NMSQuest, Non-Motor Symptoms Questionnaire; TMSE, Thai Mental State Examination; LED, levodopa equivalent dose; S&E-ADL, Schwab and England Activities of Daily Living, CVD, cerebrovascular disease; CCI, Charlson Cormorbidity Index. (DOCX) [file pone.0261302.s001.docx]

**S1 table: Comparison of demographic and clinical characteristics of Middle-Onset PD versus Late-Onset PD patient**

|  | Age of onset (yrs) | | *p-*value |
| --- | --- | --- | --- |
|  | < 70 (N = 97) | ≥ 70 (N = 55) |  |
| **Demographic variables** |  |  |  |
| Current age, yrs, mean (±SD) | 67.63 (±6.05) | 88.07 (±3.97) | <0.0001* |
| Age of PD onset, yrs, mean (±SD) | 56.68 (±5.45) | 79.02 (±5.39) | <0.0001* |
| Disease duration, yrs, mean (±SD) | 10.93 (±5.87) | 9.04 (±5.58) | 0.054 |
| Gender, male, N (%) | 58 (59.8%) | 22 (40.0%) | 0.019* |
| **Motor symptoms** |  |  |  |
| Predominant subtype, N (%) |  |  |  |
| TD | 33 (50.0%) | 30 (55.6%) | 0.544 |
| PIGD | 33 (50.0%) | 24 (44.4%) |  |
| Motor severity |  |  |  |
| UPDRS-III, mean (±SD) | 29.32 (±15.31) | 40.51 (±17.65) | <0.0001* |
| H&Y stage, mean (±SD) | 2.96 (±0.98) | 4.19 (±0.89) | <0.0001* |
| **Non motor symptoms, N (%)** |  |  |  |
| NMSQuest total, mean (±SD) | 8.69 (±3.48) | 11.34 (±2.63) | <0.0001* |
| Domain, N (%) |  |  |  |
| Gastrointestinal tract | 73 (75.3%) | 55 (100%) | <0.0001* |
| Urinary tract | 52 (53.6%) | 38 (69.1%) | 0.062 |
| Sexual function | 55 (56.7%) | 20 (36.4%) | 0.016* |
| Cardiovascular issues | 27 (27.8%) | 22 (40%) | 0.123 |
| Sleep/ fatigue | 55 (54.3%) | 38 (69.1%) | 0.132 |
| Apathy/attention/memory | 26 (26.8%) | 43 (78.2%) | <0.0001* |
| Hallucination/ delusion | 31 (32.0%) | 15 (27.3%) | 0.546 |
| Depression/ anxiety | 45 (46.4%) | 14 (25.5%) | 0.011* |
| Miscellaneous | 52 (53.6%) | 36 (65.5%) | 0.155 |
| TMSE, mean (±SD) | 26.30 (±4.88) | 17.84 (±7.69) | <0.0001* |
| **Medications** |  |  |  |
| LED, mg/d, mean (±SD) | 871.16 (±559.00) | 559.09 (±344.941) | <0.0001* |
| LED > 400 | 76 (82.6%) | 31 (51.7%) | <0.0001* |
| **Motor complication, N (%)** |  |  |  |
| Dyskinesia | 39 (40.2%) | 8 (14.5%) | 0.001* |
| Wearing-off | 48 (49.5%) | 29 (52.7%) | 0.701 |
| **Disabilities** |  |  |  |
| S&E-ADL, mean (±SD) | 75.98 (±20.70) | 48.54 (±23.91) | <0.0001* |
| Milestones, N (%) |  |  |  |
| Dementia | 12 (12.4%) | 30 (54.5%) | <0.0001* |
| Recurrent falls | 26 (26.8%) | 12 (21.8%) | 0.495 |
| Visual hallucination | 38 (39.2%) | 19 (34.5%) | 0.517 |
| Nursing home placement | 4 (4.1%) | 7 (12.7%) | 0.09 |
| Wheelchair placement | 14(14.4%) | 29(52.7%) | <0.0001* |
| Hospitalization in past year, n(%) | 16 (16.5%) | 19 (34.5%) | 0.011* |
| **Comorbidity, N (%)** |  |  |  |
| CVD | 16 (16.5%) | 19 (34.5%) | 0.011* |
| Musculoskeletal | 35 (36.1%) | 34 (61.8%) | 0.002* |
| Hypertension | 18 (18.6%) | 10 (28.2%) | 0.954 |
| Diabetes mellitus | 17 (17.5%) | 12 (21.8%) | 0.518 |
| Cancer | 2 (2.1%) | 3 (5.5%) | 0.260 |
| CCI, mean (±SD) | 1.1 (±1.21) | 2.2 (±1.19) | <0.0001* |
|  |  |  |  |

TD, tremor-dominant; PIGD, postural instability/gait difficulty; UPDRS, Unified Parkinson’s Disease Rating Scale; H&Y-S, Hoehn & Yahr staging; NMSQuest, Non-Motor Symptoms Questionnaire; TMSE, Thai Mental State Examination; LED, levodopa equivalent dose;  S&E-ADL, Schwab and England Activities of Daily Living, CVD, cerebrovascular disease; CCI, Charlson Cormorbidity Index
