# Supplementary material for: Profiling the most elderly parkinson’s disease patients: Does age or disease duration matter?
Source: PLoS One. 2021 Dec 22;16(12):e0261302. doi: 10.1371/journal.pone.0261302 (PMC8694485; doi:10.1371/journal.pone.0261302)
Supplement: S2 Table — TD, tremor-dominant; PIGD, postural instability/gait difficulty; UPDRS, Unified Parkinson’s Disease Rating Scale; H&Y-S, Hoehn & Yahr staging; NMSQuest, Non-Motor Symptoms Questionnaire; TMSE, Thai Mental State Examination; LED, levodopa equivalent dose; S&E-ADL, Schwab and England Activities of Daily Living, CVD, cerebrovascular disease; CCI, Charlson Cormorbidity Index. (DOCX) [file pone.0261302.s002.docx]

**S2 table: Comparison of demographic and clinical characteristics for younger-old PD patients with those disease duration <10 years versus those ≥10 years**

|  | Disease duration | | *p-*value |
| --- | --- | --- | --- |
|  | <10 (N = 48) | ≥ 10 (N = 44) |  |
| **Demographic variables** |  |  |  |
| Current age, yrs, mean (±SD) | 65.27 (±3.394) | 67.64 (±3.236) | 0.001* |
| Age of PD onset, yrs, mean (±SD) | 59.54 (±5.09) | 52.77 (±2.71) | <0.0001* |
| Disease duration, yrs, mean (±SD) | 5.81 (2.88) | 14.73 (±2.65) | <0.0001* |
| Gender, male, N (%) | 30 (62.5%) | 26 (59.1%) | 0.738 |
| **Motor symptoms** |  |  |  |
| Predominant subtype, N (%) |  |  |  |
| TD | 23 (47.9%) | 17 (38.6%) | 0.370 |
| PIGD | 25 (52.1%) | 27 (61.4%) |  |
| Motor severity |  |  |  |
| UPDRS-III, mean (±SD) | 20.23 (±11.11) | 36.18 (±12.66) | <0.0001* |
| H&Y, mean (±SD) | 2.51 (±0.73) | 3.25 (±0.92) | <0.0001* |
| **Non motor symptoms, N (%)** |  |  |  |
| NMSQuest total, mean (±SD) | 6.90 (±2.13) | 9.55 (±2.82) | <0.0001* |
| Domain, N (%) |  |  |  |
| Gastrointestinal | 36 (75%) | 32 (72.7%) | 0.804 |
| Urinary | 16 (33.3%) | 30 (68.2%) | 0.001* |
| Sexual | 29 (60.4%) | 24 (54.5%) | 0.569 |
| CVS | 7 (14.6%) | 18 (40.9%) | 0.005* |
| Sleep/ fatigue | 28 (58.3%) | 22 (50%) | 0.423 |
| Apathy/attention/memory | 7 (14.6%) | 13 (29.5%) | 0.082 |
| Hallucination/ delusion | 7 (14.6%) | 21 (47.7%) | 0.001* |
| Depression/ anxiety | 13 (27.1%) | 29 (65.9%) | <0.0001* |
| Miscellaneous | 27 (56.3%) | 21 (47.7%) | 0.414 |
| TMSE, mean (±SD) | 27.75 (±2.53) | 26.16 (±4.25) | 0.03* |
| **Medications** |  |  |  |
| LEDD, mg/d, mean (±SD) | 725.72 (±599.703) | 1070.86(±467.319) | 0.003* |
| **Motor complication, N (%)** |  |  |  |
| Dyskinesia | 11 (22.9%) | 26 (59.1%) | <0.0001* |
| Wearing-off | 10 (20.8%) | 34 (77.3%) | <0.0001* |
| **Disabilities** |  |  |  |
| S&E-ADL, mean (±SD) | 89 (±11.5) | 68 (±17.5) | 0.003* |
| Milestones, N (%) |  |  |  |
| Dementia | 0 (0%) | 8 (18.2%) | 0.002* |
| Recurrent falls | 7 (14.6%) | 18 (40.9%) | 0.005* |
| Visual hallucination | 10 (20.8%) | 23 (52.3%) | 0.002* |
| Nursing home placement | 0 (0%) | 3 (6.8%) | 0.066 |
| Wheelchair placement | 2 (4.2%) | 9 (20.5%) | 0.016* |
| Hospitalization in past year, N (%) | 3 (6.3%) | 11 (25%) | 0.012* |
| **Comorbidity, N (%)** |  |  |  |
| CVD | 3 (6.3%) | 11 (25%) | 0.012* |
| Musculoskeletal | 9 (18.8%) | 21 (47.7%) | 0.003* |
| Hypertension | 6 (12.5%) | 10 (22.7%) | 0.196 |
| Diabetes | 6 (23.3%) | 10 (20%) | 0.196 |
| Cancer | 1 (2.1%) | 1 (2.3%) | 0.95 |
| CCI, mean (±SD) | 0.83 (±1.08) | 1.18 (±1.24) | 0.154 |
|  |  |  |  |

TD, tremor-dominant; PIGD, postural instability/gait difficulty; UPDRS, Unified Parkinson’s Disease Rating Scale; H&Y-S, Hoehn & Yahr staging; NMSQuest, Non-Motor Symptoms Questionnaire; TMSE, Thai Mental State Examination; LED, levodopa equivalent dose;  S&E-ADL, Schwab and England Activities of Daily Living, CVD, cerebrovascular disease; CCI, Charlson Cormorbidity Index
