# Supplementary material for: Profiling the most elderly parkinson’s disease patients: Does age or disease duration matter?
Source: PLoS One. 2021 Dec 22;16(12):e0261302. doi: 10.1371/journal.pone.0261302 (PMC8694485; doi:10.1371/journal.pone.0261302)
Supplement: S3 Table — UPDRS, Unified Parkinson’s Disease Rating Scale; H&Y, Hoehn & Yahr; NMSQuest, Non-Motor Symptoms Questionnaire; TMSE, Thai Mental State Examination; LED, levodopa equivalent dose; S&E-ADL, Schwab and England Activities of Daily Living, CCI, Charlson Cormorbidity Index. a Lower scores indicate greater disability. (DOCX) [file pone.0261302.s003.docx]

**S3 Table:** Correlation of various variables with age and disease duration

| Variable | Age | | Disease duration | |
| --- | --- | --- | --- | --- |
|  | *r* | *P* | *r* | *P* |
| **UPDRS** | **0.471** | **<0.0001*** | **0.595** | **<0.0001*** |
| **HY stage** | **0.657** | **<0.0001*** | **0.402** | **0.015*** |
| **NMSQuest** | **0.553** | **<0.0001*** | **0.399** | **<0.0001*** |
| **TMSE^a^** | **0.665** | **<0.0001*** | **-0.236** | **0.003*** |
| **LED** | **-0.283** | **<0.0001*** | **0.250** | **0.002*** |
| **SE-ADL^a^** | **-0.666** | **<0.0001*** | **-0.451** | **<0.0001*** |
| **CCI** | **0.466** | **<0.0001*** | 0.144 | 0.077 |

UPDRS, Unified Parkinson’s Disease Rating Scale; H&Y, Hoehn & Yahr; NMSQuest, Non-Motor Symptoms Questionnaire; TMSE, Thai Mental State Examination; LED, levodopa equivalent dose;  S&E-ADL, Schwab and England Activities of Daily Living, CCI, Charlson Cormorbidity Index

**^a^** Lower scores indicate greater disability.
