# Supplementary material for: Profiling the most elderly parkinson’s disease patients: Does age or disease duration matter?
Source: PLoS One. 2021 Dec 22;16(12):e0261302. doi: 10.1371/journal.pone.0261302 (PMC8694485; doi:10.1371/journal.pone.0261302)
Supplement: S4 Table — Model summary. a Hosmer and Lemeshow test 0.403, Nagelkerke R square 0.099. b Hosmer and Lemeshow test 0.782, Nagelkerke R square 0.338. c Hosmer and Lemeshow test 0.406, Nagelkerke R square 0.126. d Hosmer and Lemeshow test 0.449, Nagelkerke R square 0.129. e Hosmer and Lemeshow test 0.034, Nagelkerke R square 0.044. f Hosmer and Lemeshow test 0.972, Nagelkerke R square 0.125. (DOCX) [file pone.0261302.s004.docx]

**S4 Table:** Logistic regression model for disabilities and milestones.

| **Variables** | **OR** | **95% CI** | **p-value** |
| --- | --- | --- | --- |
| **Model for Dementia ^a^** | | | |
| Age | 15.728 | 6.184 – 39.999 | <0.001* |
| Disease duration | 3.214 | 1.319 – 7.832 | 0.01* |
| **Model for wheelchair dependence ^b^** | | | |
| Age | 9.776 | 4.129 – 23.146 | <0.001* |
| Disease duration | 3.760 | 1.595 – 8.863 | 0.002* |
| **Model for Hospitalization in past 1-year ^c^** | | | |
| Age | 3.067 | 1.386 – 6.786 | 0.006* |
| Disease duration | 2.547 | 1.132 – 5.727 | 0.024* |
| **Model for nursing home placement ^d^** | | | |
| Age | 4.586 | 1.152 – 18.256 | 0.031* |
| Disease duration | 3.050 | 0.761 – 12.230 | 0.115 |
| **Model for Recurrent Fall (HY stage 1-4) ^e^** | | | |
| Age | 0.727 | 0.335 – 1.577 | 0.419 |
| Disease duration | 1.912 | 0.903 – 4.048 | 0.090 |
| **Model for Visual hallucinations ^f^** | | | |
| Age | 1.178 | 0.583 – 2.379 | 0.649 |
| Disease duration | 3.709 | 1.847 – 7.449 | <0.001* |

Model summary

a Hosmer and Lemeshow test 0.403, Nagelkerke R square 0.099

b Hosmer and Lemeshow test 0.782, Nagelkerke R square 0.338
c Hosmer and Lemeshow test 0.406, Nagelkerke R square 0.126

d Hosmer and Lemeshow test 0.449, Nagelkerke R square 0.129

e Hosmer and Lemeshow test 0.034, Nagelkerke R square 0.044

f Hosmer and Lemeshow test 0.972, Nagelkerke R square 0.125
