# Supplementary material for: Profiling the most elderly parkinson’s disease patients: Does age or disease duration matter?
Source: PLoS One. 2021 Dec 22;16(12):e0261302. doi: 10.1371/journal.pone.0261302 (PMC8694485; doi:10.1371/journal.pone.0261302)
Supplement: Attachment — Submitted filename: ResponseReviewer-PONE-D-21-03548R1-17Sep2021.docx [file pone.0261302.s006.docx]

17 September 2021

Dr. Karsten Witt

Academic Editor

PLOS ONE

Dear Dr. Witt,

Re: Manuscript # PONE-D-21-03548R1: Profiling the Most Elderly Parkinson’s Disease Patients: Does Age or Disease Duration Matter?

We are grateful to the editor and reviewers for their time and constructive comments on our manuscript. We have implemented their comments and suggestions and wish to submit a revised version of the manuscript for further consideration in the journal. Changes in the manuscript are highlighted in red in the revised version. Below, we also provide a point-by-point response explaining how we have addressed each of the editors or reviewers’ comments.

***Reviewers' comments:***

**Reviewer #1:** The authors have largely addressed my comments. However, the two groups of differing age with matched disease duration creates a major confound of age of onset in the interpretation of their results that should be addressed:

Response: Thank you for your comments. We have gone through your comments carefully and tried our best to address them one by one. We hope the manuscript has been improved accordingly.

Major comments:
The selection of participants of two age groups with matched disease duration means that the two groups differ not only in age but also in age of onset. The authors’ interpretation of the differences in the two populations as being related to age is possible, but cannot be separated, then, from the age of onset and an equally valid interpretation would be that PD patients with later age of onset (rather than old-old) are more severe, have more non motor symptoms, etc than earlier age on onset (rather than young-old), as has been shown previously. This is fundamental to the analysis and interpretation of the results and should be addressed more than simply discussing in the limitations section. The authors could match for disease duration, and age of onset separately, e.g. The subgroup analysis that, in the old old group, the longer duration (hence younger age of onset) subgroup is more severe than the shorter duration subgroup is helpful; but would not address the alternate interpretation of the differences between the young-old and old-old group.

*Response:* Thank you for raising this point. In this study, the primary objective is to fill the gap in the literature concerning the profile of old-old Parkinson’s (i.e., age ≥ 85 years old). The secondary objective is to demonstrate whether the driving force in each characteristic is worsening of the disease pathophysiology with increasing duration or age-related processes of the advanced age.

However, aside from age and disease duration, as reviewer mentioned, age at onset may have several important clinical correlations. The authors used PubMed to search relevant literature using term “Parkinson’s disease” with additional search term “late onset”. These terms were restricted to the title of the article. Duplicates were removed and all articles published in English language (n=79) were review. Among these, 37 studies have specially analyzed characteristics in patients with Late Onset PD (LOPD) compare with Middle Onset PD (MOPD), and/or Young Onset PD (YOPD) matched for disease duration. A final explanation for observe differences in these studies could related to the old age of the old-age onset cohort, independent of onset age. To test this hypothesis, our subjects composed of old-old patients matched for age at the time of data collection but with different disease duration, i.e., the longer duration (MOPD) and shorter disease duration (LOPD).

Furthermore, while there is still a lack of consensus in the age definition of early- and late-onset PD, several studies divided them into 3 distinct groups YOPD, MOPD and LOPD, and compared them with respect to the differences in clinical presentation, treatment, disease progression, biochemical profile, and nigrostriatal function. They found that the YOPD (≤ 50 years) group differs from MOPD and LOPD group in many aspects, whereas MOPD and LOPD patients are more similar (1, 2). This would imply that age of onset can contribute to the distinctive clinical-biochemical-pathological profile of YOPD from those in MOPD and LOPD, while the clinical impairment in LOPD may similarly be based in the make-up of the very old brain, including rate of nigrostriatal degeneration, reduced compensatory mechanisms, and frequency of comorbidities etc. This is also stated in previous studies (reference number 100, 101). To reflect this, and to avoid confusion due to the different pathophysiology bases of the YOPD, our studies specifically focused on MOPD and LOPD (>50 years).

To address reviewer’s concern, although we reported the results using age at examination as a predictor variable (Table 1), we repeated the analysis using age at onset of PD as a comparator and details provided in the supplementary material (see S1 table). In general, findings were similar, and based on the make-up of the very old brain as mentioned earlier. To clarify this point, we also included the following statement in the methods, result and discussion of the manuscript read as follows:

Page 7: Line 160-164

Moreover, to address the role of age at onset (AAO), we repeated the analysis in which patients were divided in 2 groups, middle-onset PD (MOPD) group included patients with AAO between 50 and 69, and the LOPD group included patients with AAO ≥70 years. This cut- off was guided by the conclusion of a cluster analysis from Post el al (3). Based on the available study subjects, we did not study those AAO of less than 50 years.

Page 19: Line 327 to Page 20: Line 353

**MOPD and LOPD: a general comparison**

Supplementary Data 1. shows details of demographic and clinical characteristics of the PD regarding to AOA. 97 patients (63%) belonged in the MOPD group and 55 (37%) in the LOPD group. Current age was 67.63 ±6.05 and 88.07 ±3.97 years in the MOPD and LOPD group, respectively (p<0.0001), whereas disease duration for both the groups was similar (10.93 vs. 9.04, *p*=0.054). Tremor or PIGD as the predominant motor symptom was not significantly different in MOPD and LOPD (p=0.544). UPDRS-III scores and H&Y stages were significantly higher in the LOPD patients compared to the MOPD patients (*p*<0.0001 and *p*<0.0001, respectively).

Considering NMS, the mean NMSQuest score was significantly greater amongst the LOPD patients than the MOPD patients (p < 0.0001). More frequently reported NMS by MOPD patients were in the domain of sexual function (*p*=0.016), followed by depression and anxiety (*p*=0.024). While features of gastrointestinal problems (*p*<0.0001) and cognitive impairment (*p*<0.0001) were significantly more reported in the LOPD patients. There were no statistically significant differences in the remaining domains.

When calculating the LEDD, the mean dosage in the MOPD patients was 559 mg and in the LOPD patients it was 871 mg (*p*<0.0001). Dyskinesia developed in 40% of the MOPD and 14.5% of the LOPD patients (p<0.001), while wearing-off rate did not differ between the groups (*p*=0.701).

For disabilities and important clinical milestones, LOPD patients were more likely to have a greater disability, as measured by the S&E-ADL scale (*p*<0.0001) and tended to have higher milestone frequency. Specific milestones more common in LOPD than in MOPD patients were dementia (*p*<0.0001), wheelchair placement (*p*<0.0001), and hospitalisation in the past year (*p*=0.011).

Page 28: Line 554 to Page 29: Line 566

This study aims to fill the gap in the literature concerning the profile of old-old Parkinson’s patients and to demonstrate whether the driving force in each characteristic is worsening of the disease pathophysiology with increasing duration or age-related processes of the advanced age. To address the role of AAO, we repeated our analysis using AAO of PD as a comparator, detailed in Supplementary data 1 and 2. In general, outcomes were similar to our other findings, as an inevitable consequence of variable-dependence around age, AAO and disease duration. Previous studies have suggested that AAO may contribute to the distinctive clinical-biochemical-pathological profile of patients with AAO ≤ 50 years (i.e., young-, early-, juvenile-onset PD; YOPD), while the different profile seen in patients with AAO > 50 years (middle- and late-onset PD) may be based on the make-up of the very old brain, including, rate of nigrostriatal degeneration, reduced compensatory mechanisms, and frequency of comorbidities (4, 5). To reflect this, and to avoid confusion due to different pathophysiology bases of YOPD, our studies specifically focused on those with AAO >50 years.

**S1 table: Comparison of demographic and clinical characteristics of Middle Onset PD versus Late Onset PD patient**

|  | Age of onset (yrs) | | *p-*value |
| --- | --- | --- | --- |
|  | < 70 (N = 97) | ≥ 70 (N = 55) |  |
| **Demographic variables** |  |  |  |
| Current age, yrs, mean (±SD) | 67.63 (±6.05) | 88.07 (±3.97) | <0.0001* |
| Age of PD onset, yrs, mean (±SD) | 56.68 (±5.45) | 79.02 (±5.39) | <0.0001* |
| Disease duration, yrs, mean (±SD) | 10.93 (±5.87) | 9.04 (±5.58) | 0.054 |
| Gender, male, N (%) | 58 (59.8%) | 22 (40.0%) | 0.019* |
| **Motor symptoms** |  |  |  |
| Predominant subtype, N (%) |  |  |  |
| TD | 33 (50.0%) | 30 (55.6%) | 0.544 |
| PIGD | 33 (50.0%) | 24 (44.4%) |  |
| Motor severity |  |  |  |
| UPDRS-III, mean (±SD) | 29.32 (±15.31) | 40.51 (±17.65) | <0.0001* |
| H&Y stage, mean (±SD) | 2.96 (±0.98) | 4.19 (±0.89) | <0.0001* |
| **Non motor symptoms, N (%)** |  |  |  |
| NMSQuestUEST total, mean (±SD) | 8.69 (±3.48) | 11.34 (±2.63) | <0.0001* |
| Domain, N (%) |  |  |  |
| Gastrointestinal tract | 73 (75.3%) | 55 (100%) | <0.0001* |
| Urinary tract | 52 (53.6%) | 38 (69.1%) | 0.062 |
| Sexual function | 55 (56.7%) | 20 (36.4%) | 0.016* |
| Cardiovascular issues | 27 (27.8%) | 22 (40%) | 0.123 |
| Sleep/ fatigue | 55 (54.3%) | 38 (69.1%) | 0.132 |
| Apathy/attention/memory | 26 (26.8%) | 43 (78.2%) | <0.0001* |
| Hallucination/ delusion | 31 (32.0%) | 15 (27.3%) | 0.546 |
| Depression/ anxiety | 45 (46.4%) | 14 (25.5%) | 0.011* |
| Miscellaneous | 52 (53.6%) | 36 (65.5%) | 0.155 |
| TMSE, mean (±SD) | 26.30 (±4.88) | 17.84 (±7.69) | <0.0001* |
| **Medications** |  |  |  |
| LED, mg/d, mean (±SD) | 871.16 (±559.00) | 559.09 (±344.941) | <0.0001* |
| LED > 400 | 76 (82.6%) | 31 (51.7%) | <0.0001* |
| **Motor complication, N (%)** |  |  |  |
| Dyskinesia | 39 (40.2%) | 8 (14.5%) | 0.001* |
| Wearing-off | 48 (49.5%) | 29 (52.7%) | 0.701 |
| **Disabilities** |  |  |  |
| S&E-ADL, mean (±SD) | 75.98 (±20.70) | 48.54 (±23.91) | <0.0001* |
| Milestones, N (%) |  |  |  |
| Dementia | 12 (12.4%) | 30 (54.5%) | <0.0001* |
| Recurrent falls | 26 (26.8%) | 12 (21.8%) | 0.495 |
| Visual hallucination | 38 (39.2%) | 19 (34.5%) | 0.517 |
| Nursing home placement | 4 (4.1%) | 7 (12.7%) | 0.09 |
| Wheelchair placement | 14(14.4%) | 29(52.7%) | <0.0001* |
| Hospitalization in past year, n(%) | 16 (16.5%) | 19 (34.5%) | 0.011* |
| **Comorbidity, N (%)** |  |  |  |
| CVD | 16 (16.5%) | 19 (34.5%) | 0.011* |
| Musculoskeletal | 35 (36.1%) | 34 (61.8%) | 0.002* |
| Hypertension | 18 (18.6%) | 10 (28.2%) | 0.954 |
| Diabetes mellitus | 17 (17.5%) | 12 (21.8%) | 0.518 |
| Cancer | 2 (2.1%) | 3 (5.5%) | 0.260 |
| CCI, mean (±SD) | 1.1 (±1.21) | 2.2 (±1.19) | <0.0001* |
|  |  |  |  |

Furthermore, our study extended the analysis to the young-old with short and long disease duration, however, as to available study subjects, there were significant different in both age of onset and age at examination. See the supplementary material (S2 table) We also included the following statement in the methods and result section as following.

Page 7: Line 160-164

To investigate the influence of disease duration, patients of the old-old and young-old groups were also separated into two subgroups, according to duration of illness, into a ‘less than 10 years’ group, called the short duration group, and an ‘equal to or more than 10 years’ group, called the long duration group, based on a commonly used cut-off point from previously published studies (6, 7).

Page 20: Line 356-358

Our study extended these comparisons to the young-old patients with short and long disease duration. However, as to the available subjects, there was a significant different in age at examination (Supplementary Data 2).

**S2 table: Comparison of demographic and clinical characteristics for younger-old PD patients with those disease duration <10 years versus those ≥10 years**

|  | Disease duration | | *p-*value |
| --- | --- | --- | --- |
|  | <10 (N = 48) | ≥ 10 (N = 44) |  |
| **Demographic variables** |  |  |  |
| Current age, yrs, mean (±SD) | 65.27 (±3.394) | 67.64 (±3.236) | 0.001* |
| Age of PD onset, yrs, mean (±SD) | 59.54 (±5.09) | 52.77 (±2.71) | <0.0001* |
| Disease duration, yrs, mean (±SD) | 5.81 (2.88) | 14.73 (±2.65) | <0.0001* |
| Gender, male, N (%) | 30 (62.5%) | 26 (59.1%) | 0.738 |
| **Motor symptoms** |  |  |  |
| Predominant subtype, N (%) |  |  |  |
| TD | 23 (47.9%) | 17 (38.6%) | 0.370 |
| PIGD | 25 (52.1%) | 27 (61.4%) |  |
| Motor severity |  |  |  |
| UPDRS-III, mean (±SD) | 20.23 (±11.11) | 36.18 (±12.66) | <0.0001* |
| H&Y, mean (±SD) | 2.51 (±0.73) | 3.25 (±0.92) | <0.0001* |
| **Non motor symptoms, N (%)** |  |  |  |
| NMSQuest total, mean (±SD) | 6.90 (±2.13) | 9.55 (±2.82) | <0.0001* |
| Domain, N (%) |  |  |  |
| Gastrointestinal | 36 (75%) | 32 (72.7%) | 0.804 |
| Urinary | 16 (33.3%) | 30 (68.2%) | 0.001* |
| Sexual | 29 (60.4%) | 24 (54.5%) | 0.569 |
| CVS | 7 (14.6%) | 18 (40.9%) | 0.005* |
| Sleep/ fatigue | 28 (58.3%) | 22 (50%) | 0.423 |
| Apathy/attention/memory | 7 (14.6%) | 13 (29.5%) | 0.082 |
| Hallucination/ delusion | 7 (14.6%) | 21 (47.7%) | 0.001* |
| Depression/ anxiety | 13 (27.1%) | 29 (65.9%) | <0.0001* |
| Miscellaneous | 27 (56.3%) | 21 (47.7%) | 0.414 |
| TMSE, mean (±SD) | 27.75 (±2.53) | 26.16 (±4.25) | 0.03* |
| **Medications** |  |  |  |
| LEDD, mg/d, mean (±SD) | 725.72 (±599.703) | 1070.86(±467.319) | 0.003* |
| **Motor complication, N (%)** |  |  |  |
| Dyskinesia | 11 (22.9%) | 26 (59.1%) | <0.0001* |
| Wearing-off | 10 (20.8%) | 34 (77.3%) | <0.0001* |
| **Disabilities** |  |  |  |
| S&E-ADL, mean (±SD) | 89 (±11.5) | 68 (±17.5) | 0.003* |
| Milestones, N (%) |  |  |  |
| Dementia | 0 (0%) | 8 (18.2%) | 0.002* |
| Recurrent falls | 7 (14.6%) | 18 (40.9%) | 0.005* |
| Visual hallucination | 10 (20.8%) | 23 (52.3%) | 0.002* |
| Nursing home placement | 0 (0%) | 3 (6.8%) | 0.066 |
| Wheelchair placement | 2 (4.2%) | 9 (20.5%) | 0.016* |
| Hospitalization in past year, N (%) | 3 (6.3%) | 11 (25%) | 0.012* |
| **Comorbidity, N (%)** |  |  |  |
| CVD | 3 (6.3%) | 11 (25%) | 0.012* |
| Musculoskeletal | 9 (18.8%) | 21 (47.7%) | 0.003* |
| Hypertension | 6 (12.5%) | 10 (22.7%) | 0.196 |
| Diabetes | 6 (23.3%) | 10 (20%) | 0.196 |
| Cancer | 1 (2.1%) | 1 (2.3%) | 0.95 |
| CCI, mean (±SD) | 0.83 (±1.08) | 1.18 (±1.24) | 0.154 |
|  |  |  |  |

TD, tremor-dominant; PIGD, postural instability/gait difficulty; UPDRS, Unified Parkinson’s Disease Rating Scale; H&Y-S, Hoehn & Yahr staging; NMSQuest, Non-Motor Symptoms Questionnaire; TMSE, Thai Mental State Examination; LED, levodopa equivalent dose;  S&E-ADL, Schwab and England Activities of Daily Living, CVD, cerebrovascular disease; CCI, Charlson Cormorbidity Index

Minor comments:

Line 72: grammatical error needing clarification, are there 13 million elderly adults comprising 20% of the population?

Response:

- We have corrected this grammatical error in line 72.
- The data source was the “Policy Mapping on Ageing in Asia and the Pacific Analytical Report”. We already provided the following citations to support this statement (Reference number 4)

4. HelpAge International. 2015. Policy Mapping on Ageing in Asia and the Pacific Analytical Report. Chiang Mai: HelpAge International East Asia/Pacific Regional Office.

Reviewer #2: Generally a satisfactory revision.

My Point 5: I think the subtyping criteria should be clearly stated in METHODS, not just referenced elsewhere

Response: Thank you for your nice reminder. We already provided an explanation in METHODS, Subsection Measurement of clinical variables as following (see page 8, line 174-179).

“PD motor subtypes were identified following the original classification methods into two subtypes: (1) tremor- dominant PD and (2) postural instability and gait difficulty (PIGD), using the UPDRS, an average global tremor score and a mean score for the complex of PIGD were determined, and patients were assigned to a tremor group and a PIGD group based on the ratio of these scores (8, 9).”

I would like to confirm that all authors have read the manuscript; the paper has not been previously published, and is not under simultaneous consideration by another journal. There is also no ghost writing by anyone not named on the author list.

There is no conflict of interest on all authors and we will take full responsibility for the data, the analyses and interpretation, and the conduct of the research. We had full access to all of the data; and that we had the right to publish any and all data, separate and apart from the attitudes of the sponsor.

Thank you very much for consideration our manuscript for publication. Please let me know if there are any questions.

We are grateful to the editors and reviewers for the time and effort that they have put into helping us improve our manuscript.

Sincerely,

Corresponding author:

Roongroj Bhidayasiri, MD., FRCP., FRCPI.

Chulalongkorn Center of Excellence on Parkinson Disease and Related Disorders

Chulalongkorn University Hospital

1873 Rama 4 Road

Bangkok 10330

Thailand

Tel: +662-256-4000 ext. 70701

Fax: +662-256-4630

Email address: rbh@chulapd.org

**References**

1.	Mehanna R, Moore S, Hou JG, Sarwar AI, Lai EC. Comparing clinical features of young onset, middle onset and late onset Parkinson's disease. Parkinsonism Relat Disord. 2014;20(5):530-4.

2.	Schirinzi T, Di Lazzaro G, Sancesario GM, Summa S, Petrucci S, Colona VL, et al. Young-onset and late-onset Parkinson's disease exhibit a different profile of fluid biomarkers and clinical features. Neurobiol Aging. 2020;90:119-24.

3.	Post B, Speelman JD, de Haan RJ. Clinical heterogeneity in newly diagnosed Parkinson's disease. J Neurol. 2008;255(5):716-22.

4.	Diederich NJ, Moore CG, Leurgans SE, Chmura TA, Goetz CG. Parkinson Disease With Old-Age Onset: A Comparative Study With Subjects With Middle-Age Onset. Archives of Neurology. 2003;60(4):529-33.

5.	Levy G, Louis ED, Cote L, Perez M, Mejia-Santana H, Andrews H, et al. Contribution of aging to the severity of different motor signs in Parkinson disease. Arch Neurol. 2005;62(3):467-72.

6.	Hely MA, Morris JGL, Traficante R, Reid WGJ, O’Sullivan DJ, Williamson PM. The Sydney multicentre study of Parkinson’s disease: progression and mortality at 10 years. Journal of Neurology, Neurosurgery &amp;amp; Psychiatry. 1999;67(3):300.

7.	Hassan A, Wu SS, Schmidt P, Malaty IA, Dai YF, Miyasaki JM, et al. What are the issues facing Parkinson's disease patients at ten years of disease and beyond? Data from the NPF-QII study. Parkinsonism Relat Disord. 2012;18 Suppl 3:S10-4.

8.	Stebbins GT, Goetz CG, Burn DJ, Jankovic J, Khoo TK, Tilley BC. How to identify tremor dominant and postural instability/gait difficulty groups with the movement disorder society unified Parkinson's disease rating scale: comparison with the unified Parkinson's disease rating scale. Mov Disord. 2013;28(5):668-70.

9.	Thenganatt MA, Jankovic J. Parkinson Disease Subtypes. JAMA Neurology. 2014;71(4):499-504.
